# Recurrent Extreme Climatic Events Are Driving Gorgonian Populations to Local Extinction: Low Adaptive Potential to Marine Heatwaves

**DOI:** 10.1111/gcb.17587

**Published:** 2024-11-27

**Authors:** Sandra Ramirez‐Calero, Daniel Gómez‐Gras, Aldo Barreiro, Nathaniel Bensoussan, Laura Figuerola‐Ferrando, Marc Jou, Àngel López‐Sanz, Paula López‐Sendino, Alba Medrano, Ignasi Montero‐Serra, Marta Pagès‐Escolà, Cristina Linares, Jean‐Baptiste Ledoux, Joaquim Garrabou

**Affiliations:** ^1^ Departament de Biologia Marina Institut de Ciències del Mar (CSIC) Barcelona Spain; ^2^ Departament de Biologia Evolutiva, Ecologia i Ciències Ambientals Universitat de Barcelona (UB) Barcelona Spain; ^3^ Institut de Recerca de la Biodiversitat (IRBio) Universitat de Barcelona Barcelona Spain; ^4^ CIIMAR/CIMAR, Centro Interdisciplinar de Investigação Marinha e Ambiental Universidade do Porto Porto Portugal; ^5^ CNRS, IRD, Mediterranean Institute of Oceanography (MIO) UTM 110, University of Toulon University of Aix‐Marseille Marseilles France; ^6^ CNRS, Ifremer, IRD, Laboratoire d'Océanographie Physique et Spatiale (LOPS), IUEM Univ Brest Plouzané France

**Keywords:** common‐garden experiment, ecological memory, genotype‐by‐environment interactions, habitat‐forming octocorals, mass mortality events, Mediterranean Sea

## Abstract

Extreme climatic events (ECEs), such as marine heatwaves (MHWs), are a major threat to biodiversity. Understanding the variability in ecological responses to recurrent ECEs within species and underlying drivers arise as a key issue owing to their implications for conservation and population recovery. Yet, our knowledge on such ecological responses is limited since it has been frequently gathered following “single‐event approaches” focused on one particular event. These approaches provide snapshots of ecological responses but fall short of capturing heterogeneity patterns that may occur among recurrent ECEs, questioning current predictions regarding biodiversity trends. Here, we adopt a “multiple events” perspective to characterize the effects of recurrent ECEs on the ecological responses in *Paramuricea clavata* , a Mediterranean temperate coral threatened by MHWs. Through a common‐garden experiment repeated three consecutive years with the same individuals from three populations, we assessed the respective roles of environmental (year effect), genetic (population effect), and phenotypic (population‐by‐environment interactions effect) components in the ecological response to recurrent heat stress. The environmental component (year) was the main driver underlying the responses of *P*. *clavata* colonies across experiments. To build on this result, we showed that: (i) the ecological responses were not related to population (genetic isolation) and individual (multilocus heterozygosity) genetic make‐up, (ii) while all the individuals were characterized by a high environmental sensitivity (genotype‐by‐environment interactions) likely driven by in situ summer thermal regime. We confront our experimental results to in situ monitoring of the same individuals conducted in 2022 following two MHWs (2018, 2022). This confirms that the targeted populations harbor limited adaptive and plastic capacities to on‐going recurrent ECEs and that *P*. *clavata* might face unavoidable population collapses in shallow Mediterranean waters. Overall, we underscore the need to consider the recurrence of ECEs to assess threats to biodiversity and to forecast its evolution.

## Graphical abstract

The impact of extreme climatic events (ECEs) on biodiversity has been mostly studied focusing on one particular event at a time. This “single‐event approach” falls short of capturing the temporal heterogeneity in ecological responses occurring among ECEs. Adopting a “multiple events” perspective, we combined repeated common‐garden experiments and population genetics to characterize the effects of recurrent marine heatwaves (MHWs) in the Mediterranean octocoral, *Paramuricea clavata* . We demonstrate (i) the major influence of summer thermal regime on the responses to thermal stress and (ii) the low adaptive potential, whether genetic or plastic, of the species to recurrent MHWs.

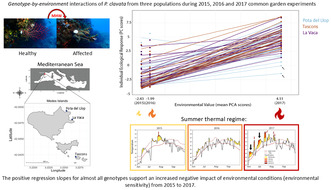

## 1 Introduction

Extreme climatic events (ECEs) linked to climate change such as heatwaves pose significant challenges for biodiversity (Jacox et al. 2020; Maxwell et al. 2019; Parmesan, Root, and Willig 2000; Pinsky et al. 2019; Ummenhofer and Meehl 2017). ECEs have been associated with the increased frequency of mass mortality events (MMEs), accelerating species demographic decline (Bramanti et al. 2005; Leung, Connell, and Russell 2017; Smale and Wernberg 2013) and questioning the future of biodiversity (Smale et al. 2019). Besides demographic decline, field surveys revealed heterogeneity in the patterns of ecological responses (sensu Maxwell et al. 2019) to ECEs across taxonomic (species, populations, and individuals), spatial (regions), and temporal (years) scales (van Bergen et al. 2020; Hughes et al. 2003; Pansch et al. 2018). Yet, to date, the potential effects of recurrent ECEs and the related temporal variability in ecological responses have been poorly considered (but see Ahrens et al. 2021; Brown et al. 2023; Brown and Barott 2022; Husson et al. 2022; Logan et al. 2014; Regan and Sheldon 2023). Indeed, most of our knowledge on ecological responses to ECEs has been gathered studying one particular event following a “single‐event approach” (Altwegg et al. 2017). This approach can provide an informative snapshot of the ecological responses to ECE but falls short of revealing the consequences of recurrent ECEs on biodiversity. Accordingly, the need to develop a “multiple events” perspective has been recently acknowledged to improve predictions on species abilities to face ECEs (Bailey and Van De Pol 2016).

Ecological responses to ECEs, considered here as phenotypes, are shaped by the interplay among “genetic,” “environmental,” and “plastic” components (Merilä and Hendry 2014). This interplay has been investigated using common‐garden experiments (Malyshev et al. 2016) and long‐term time series (Regan and Sheldon 2023). The “genetic” component relies on the standing genetic variation (Dixon et al. 2015), shaped by the balance between evolutionary forces (e.g., local adaptation, genetic drift; Bay and Palumbi 2014). The “environmental” component includes biotic (e.g., species interactions) and abiotic (e.g., temperature) factors that characterize a specific habitat and influence ecological responses (Scheiner 1993). Finally, “plastic” components result from the interaction of genetic and environmental components. These “population‐by‐environment” and “genotype‐by‐environment” interactions underlie plasticity in phenotypic response at the population and individual levels (Matesanz and Ramírez‐Valiente 2019). Individual phenotypic plasticity, considered as a single genotype expressing different phenotypes function of the environment, can provide a short‐term buffer allowing organisms to immediately face ECEs before genetic adaptation occurs (Chevin, Lande, and Mace 2010; Reusch 2014).

Marine heatwaves (MHWs), known as discrete periods of anomalously warm water (Frölicher, Fischer, and Gruber 2018; Smith et al. 2023), are some of the most challenging ECEs for marine diversity, threatening ecosystem's structure and functioning (Smale et al. 2019) from kelp forests (e.g., Arafeh‐Dalmau et al. 2020) to coral communities (e.g., Genin et al. 2020; Gómez‐Gras, Linares, Dornelas, et al. 2021; Hughes et al. 2021). In the last two decades, the Mediterranean Sea has been recurrently impacted by MHWs driving mass mortality events (MMEs) affecting multiple phyla of benthic macroinvertebrates (Cramer et al. 2018; Garrabou et al. 2022). In this particular case, as in other marine ecosystems, field surveys (e.g., Garrabou et al. 2001) and long‐term monitoring studies (Gómez‐Gras, Linares, López‐Sanz, et al. 2021; Iborra et al. 2022; Montero‐Serra et al. 2019; Ruffaldi Santori et al. 2021; Santangelo et al. 2015) combined with “single‐event” experiments in controlled conditions (Crisci et al. 2017; Gómez‐Gras et al. 2022), provided insights into the heterogeneity of ecological responses (level of tissue necrosis) within species impacted by MHWs. Yet, whatever the system considered, the impact of genetic, environmental, and plastic components underlying the differential ecological responses remain poorly understood, particularly, in the context of recurrent MHWs (but see Hughes et al. 2021).

We aim to advance the characterization of the impacts of recurrent ECEs on within‐species diversity and to infer the respective roles of the genetic, environmental, and plastic components in the ecological responses. We adopt a multiple events perspective combining experiments and in situ survey focusing on the Mediterranean scene and on the red gorgonian *Paramuricea clavata* (Risso et al. 1826). This habitat‐forming octocoral is a well‐suitable candidate given the impacts of MHWs on shallow populations monitored for 20 years (Garrabou et al. 2021, see below). We repeated during three consecutive years (2015, 2016, and 2017) a common‐garden thermal stress experiment at fine spatial scale (populations separated by < 1 km), in which we controlled for different aspects of genetic (same genotypes tested) and environmental (same experimental set‐up) components. Specifically, we (i) computed the percentage of heterogeneity in the ecological responses (estimated based on the level of tissue necrosis), respectively, explained by the taxonomic (individual and populations) and temporal (years) variabilities; (ii) tested the significance of the genetic (population effect), environmental (year effect), and plastic (population‐by‐years effect) components on such ecological responses. We discussed the obtained results in the light of (iii) the populations and individuals' genetic make‐up (i.e., genetic drift and heterosis); (iv) environmental sensitivity analyses looking at genotype‐by‐environment interactions; and (v) in situ summer thermal regimes. We (vi) contrasted the ecological responses from the experiments to the ecological responses reported from a field survey of the same individuals conducted in 2022 after two MHWs.

## 2 Materials and Methods

### 2.1 Species of Interest

The red gorgonian *P*. *clavata* is a habitat‐forming octocoral with an important role in the structure and functioning of the Mediterranean coralligenous habitats (Boavida et al. 2016; Gómez‐Gras, Linares, Dornelas, et al. 2021; Ponti et al. 2014, 2018). This species displays low population dynamics with slow growth, recruitment, and recovery rates and late sexual maturity (Gómez‐Gras, Linares, Dornelas, et al. 2021; Gómez‐Gras, Linares, López‐Sanz, et al. 2021, but see Cupido et al. 2009), as well as restricted dispersal abilities resulting in a reduced resilience (Coma et al. 1998; Ledoux et al. 2018; Linares et al. 2008; Mokhtar‐Jamaï et al. 2011). *Paramuricea clavata* has been particularly impacted by recurrent marine heatwaves in the past 20 years (Cebrian et al. 2012; Garrabou et al. 2021; Gómez‐Gras, Linares, López‐Sanz, et al. 2021). It was included in the IUCN red list of vulnerable Mediterranean Anthozoans (Otero et al. 2017). “Single‐event” experiments using common‐garden set‐ups identified the thermal risk zone of this species for temperatures over 25°C (Crisci et al. 2017). These experiments have characterized the ecological responses (tissue necrosis) and underlying processes in populations from contrasted environments (depth) and separated by distances of tens to thousands of kilometers (Arizmendi‐Mejia, Linares, et al. 2015; Arizmendi‐Mejia, Ledoux, et al. 2015; Crisci et al. 2017; Gómez‐Gras et al. 2022). Altogether, the ecological importance of *P*. *clavata* as a habitat‐forming species and the extensive available ecological information make this model highly relevant to evaluate the roles of the genetic, environmental, and plastic components on the ecological responses to recurrent ECEs.

### 2.2 Identification of the Colonies

**FIGURE 1:**
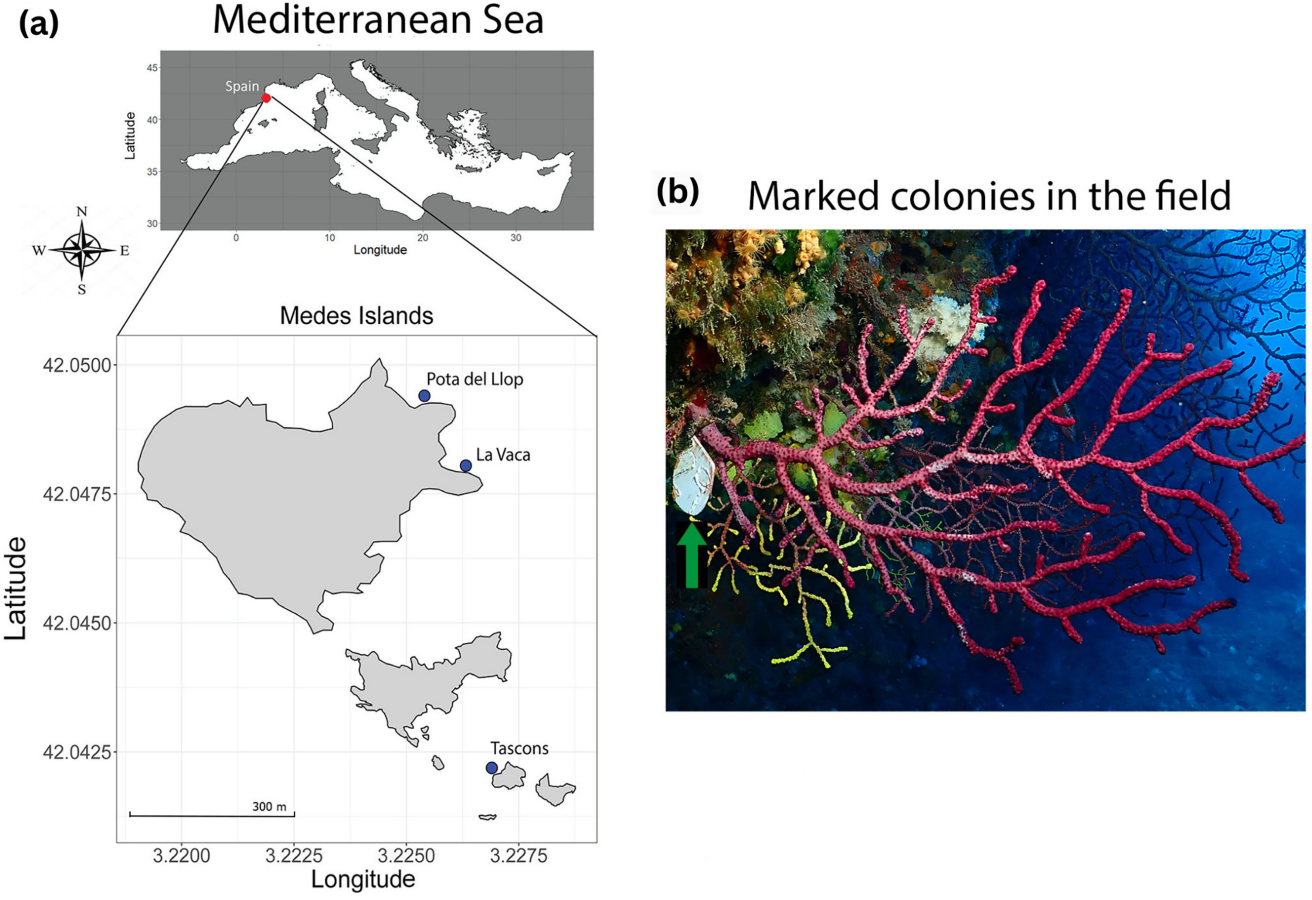
(a) *Paramuricea clavata* sampling sites at Medes Islands, Spain. (b) Tagged colony of *P*. *clavata* in Tascon's location (green arrow). Map lines delineate study areas and do not necessarily depict accepted national boundaries.

Overall 90 adult colonies (> 50 cm) were randomly chosen around 15 m depth from three different sites (30 colonies each), separated by hundreds of meters at Medes Islands, Spain (42°02′60.00″ N 3°12′60.00″ E): Pota del Llop (*N* = 30), La Vaca (*N* = 30), and Tascons (*N* = 30, Figure 1a). Each colony was marked during September of 2014 using plastic tags with a unique ID (Figure 1b). From every marked colony, two apical fragments of 10 cm were collected between September 21 and October 22 of 2015, 2016, and 2017. These fragments were retrieved in 2 L bags of water and immediately transported (maximum transportation time 2 h) in coolers to the Aquarium Experimental Zone (ZAE) of the Institut de Ciències del Mar (ICM‐CSIC, Barcelona, Spain).

### 2.3 Common‐Garden Set‐Up Repeated in 2015, 2016, and 2017

Upon arrival, colony fragments were mechanically fixed to experimental plates as described in Crisci et al. (2017) (Figure S1). All colonies were acclimated for 1 week in an open water system with 50 μm sand‐filtered running seawater at 17°C–18°C. No mortality signs and tissue necrosis were detected during this period in any of the colonies and/or years. Moreover, all sampled colonies showed active polyps during feeding. After the acclimation period, we conducted the common‐garden experiments as described in Crisci et al. (2017). Specifically, each fragment was divided into two subfragments, one for control (18°C) and one for the heat stress treatment (25°C). For the heat stress treatment, temperature was increased from 18°C to 25°C over a period of 3 days and maintained at 25°C for the next 28 days. Colonies were fed during the entire experimental course (see Data S1 for further details about the set up).

### 2.4 Phenotypic Response: Survival Analysis, Individual Fitness, and Modeling the Response to Thermal Stress

Several descriptors were used to statistically compare the ecological responses among colonies and populations. The percentage of tissue necrosis (extent of injury) by colony was monitored visually every day considering 5% step intervals. Ecological impacts on *P*. *clavata* were considered as “low” when 10%–30% necrosis was observed, “moderate” when necrosis was > 30%–60%, and “severe” for > 60% necrosis, following the impact classification of the T‐MEDNet mass mortality database (e.g., Garrabou et al. 2019). In addition, we estimated for each population and year: (i) the daily extent of injury per colony (% of tissue necrosis), (ii) the daily percentage of affected colonies (> 10% of tissue necrosis), and (iii) the daily percentage of dead colonies (100% tissue necrosis).

In spite of the plastic tags, we were not able to retrieve and sample all the 90 individuals for the three common‐garden experiments. Accordingly, the following statistical analyses account for the individuals that were at least included in two of the three common gardens, resulting in 29 individuals from “Tascons,” 28 from “Pota del Llop,” and 19 from “La Vaca.” To model the response to thermal stress across years and populations, we computed a principal component analysis (PCA) with individuals and years as cases and the percentage of necrosis per day as variables. A first exploratory PCA explained 50.29% of the variance and was strongly correlated with the percentage of necrosis from days 10 to 28 (Figure S2a). To improve the fitness of the data, days 1 to 9 were removed from the dataset and a second PCA was conducted. This second PCA explained 75% of the variance for the remaining days (Figure S2b). All PCAs were performed with *PCA*() function from FactoMineR R package (R Core Team 2022; Lê, Josse, and Husson 2008). Scores from the PC1 from the second PCA were considered as a proxy for individual fitness as they are directly related to the development of necrosis across the 10th to the 28th days. These PC scores were employed as response variable for two linear models: (1) including “population” and “year” as predictor variables (i.e., fixed factors); (2) using “individual” as a random factor, added to the previous predictor variables, and affecting only the model intercepts. Available data were not sufficient to fit all the extra number of parameters (i.e., coefficients), thus the effect of the factor “individual” was not tested in the slope of the model. We consider the “individual” effect in the intercept as a different baseline of resistance to thermal stress. Linear models were fitted using *lm*() and *lmer*() functions from the stats and the *lme4*() R packages (Fox and Weisberg 2019). To meet the assumptions of residual normality and homoscedasticity, we transformed the response variable with the Box–Cox transformation, implemented with the *boxcox*() function from *MASS* R package (Venables and Ripley 2002). These assumptions were tested with the Shapiro–Wilks and Levene tests using *Shapiro.test*() and *leveneTest*() from *car* R package (Fox and Weisberg 2019). The best model was selected based on AIC (Akaike's information criterion) using the function *anova*() (R Core Team 2022) and a post hoc Tukey test with the *glht*() function from *multcomp* R package (Hothorn, Bretz, and Westfall 2008). We estimated the percentage of the contribution of each factor to the total variance computing the difference in log‐likelihood between models with and without each factor (individual, population, and year) pondered by the degrees of freedom. The random intercepts obtained for every individual in the linear model 2 (hereafter “nec‐int”) were used as a proxy for individual fitness to test for heterosis (see below). Finally, independence and randomness were maintained in all tests, as the only structure of dependence existing in the design was created by the individuals, considered here as a random factor.

### 2.5 Genetic Components: Population Structure and Individual Heterosis

DNA extractions, genotyping protocols, microsatellite characteristics, quality check, and analyses of genetic diversity are described in Data S1. We characterized the genetic diversity and structure of the three sampling sites by genotyping 87 individuals collected in at least two of the 3 years with 14 microsatellites (accession number: GU386255–GU386265 and PQ513430–PQ513439; Agell, Rius, and Pascual 2009; Aurelle et al. 2010; Ledoux et al. 2018) (see Data S1 for additional details).

We conducted a discriminant analysis of principal components (DAPC; Jombart, Devillard, and Balloux 2010) from *adegenet* R package (Jombart, Devillard, and Balloux 2010) by implementing the function *find_clusters*() and specifying a maximum number of clusters with *max.n.clust* = 3. A maximum number of 100 PCs was chosen and the lowest value of Bayesian information criterion (BIC) was applied to estimate the number of clusters. GENEPOP 4.1.4 (Rousset 2008) was used to compute the overall and pairwise *F*_STs_ estimators from Weir and Cockerham (1984). Genotypic differentiation between sites was tested using an exact test (Raymond and Rousset 1995) with default parameters. In small and isolated populations, inbreeding depression can negatively impact individual fitness constraining the response to ECEs (Fitzpatrick and Reid 2019). Accordingly, we estimated the genetic differentiation proper to each site by calculating the site‐specific *F*_ST_ and 95% high‐probability density intervals in GESTE (Foll and Gaggiotti 2006). The site‐specific *F*_ST_ estimates the relative impact of genetic drift on the differentiation of the considered site relative to the remaining ones.

At the individual level, a positive correlation between heterozygosity and fitness‐related traits, known as heterosis, has been reported in some species (David 1997). We looked for heterosis in the response to thermal stress testing the correlation between the values of *nec‐int* as proxy for individual fitness and the standardized individual multilocus heterozygosities (*sMLH*) computed using the R package *InbreedR*() (Stoffel et al. 2016). The slope of the linear model (*lm*[*nec‐int ~ sMLH*]) was compared to its null distribution obtained with a Monte Carlo permutation test with 10,000 permutations.

### 2.6 Environmental Component: Summer Thermal Environments

We analyzed and compared the in situ thermal regime at 15 m depth experienced by the colonies before sampling. Temperature data for Medes islands was obtained from the T‐MEDNet database (Jou and Ramirez‐Calero 2024), which follows a continuous temperature series since 2004 (von Schuckmann et al. 2019). Data were adjusted for our study (Ramirez‐Calero 2024). We assessed the recent summer local thermal regime calculated over the 3 months period of June, July, and August, prior to collection in September. We consider the timing and magnitude of the daily temperature cycles and the exposure to warm conditions; thus, we consider the averages of maximum temperatures during the warmest periods of the year, number of days with high temperatures, and the ecological threshold T23 (i.e., number of extreme heat days at ≥ 23°C). In addition, we considered positive temperature anomalies as the number of marine heat spikes (MHS) above the interannual percentile 90th (iT90 threshold) lasting less than 5 days, while prolonged discrete periods of anomalously warm water surpassing the iT90 percentile for more than 5 days, were considered as MHWs. Presence of MHWs were detected with Python module *marineHeatWaves* (Hobday et al. 2016), while impact categories registered at Medes islands were assigned following the classification of the T‐MEDNet database (Jou and Ramirez‐Calero 2024; Garrabou et al. 2019; Hobday et al. 2018). MHWs categories were set as significant temperature fold changes from the interannual mean temperature and the iT90 threshold. Thus, “Low” classifications correspond to the temperature ranges between the interannual mean temperatures and iT90, while “Moderate” are considered as a one‐ to twofold, “Strong” as a two‐ to threefold, and “Severe” as a three‐ to fourfold, respectively.

### 2.7 Plastic Component: “Genotype‐By‐Environment” Interactions and Environmental Sensitivity

We estimated the variability in the “genotype‐by‐environment” interactions by characterizing the environmental sensitivity of each genotype following Falconer and Mackay (1996). We computed three environmental values corresponding to the yearly mean phenotypes (i.e., mean PC scores over individuals for each year), considering the 76 genotypes present during the 3 years. We then plotted each individual phenotype (PC score) against the environmental value for each year and we computed the regression slope, which is considered as an estimator of the environmental sensitivity of the genotype (Falconer and Mackay 1996). We used this plot to classify the sensitivity of the genotypes in three categories adapted from Bonacolta et al. (2024). Resistant genotypes were expected to show low intercepts in the first year and approximated null slopes (i.e., low and constant level of necrosis in the three experiments). Hypersensitive genotypes were expected to show high intercepts in the first year and null slopes (i.e., high and constant level of necrosis in the three experiments). Finally, sensitive genotypes were expected to show low intercepts in the first year and positive slopes (i.e., increasing level of necrosis through time). The significance of the slope for each individual was tested with a comparison with a null distribution of regression slopes. This null distribution was obtained from 10,000 randomizations of the original data. These randomizations were made by assigning each individual PC score and environmental values randomly and fitting the slopes for the regressions on the resulting 10,000 groups of three data points.

### 2.8 Field Survey of Necrosis Rates Following 2018–2022 MMEs

Medes Islands were impacted by two MHWs in 2018 and 2022 with associated mass mortality events (Garrabou et al. 2022; Zentner et al. 2023). We surveyed by scuba diving the percentage of tissue necrosis in situ for the same individuals used in the experiments. This survey was done in October 2022. Differences in mean tissue necrosis of colonies between populations were tested using a parametric one‐way ANOVA, followed by post hoc Tukey's HSD tests.

## 3 Results

### 3.1 Phenotypic Responses of *P*. *clavata* During the Three Common‐Garden Experiments

**FIGURE 2:**
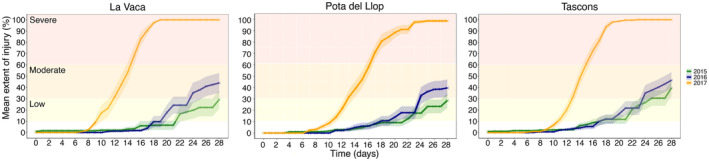
Average tissue necrosis (mean extent of injury ± SE) of *Paramuricea clavata* colonies during the 28 days of exposure in common‐garden experiments for La Vaca, Pota del Llop, and Tascons in 2015 (green), 2016 (blue), and 2017 (yellow). Necrosis severity is highlighted in light yellow (low), orange (moderate), and red (severe).

Signs of tissue necrosis were observed for all populations in the 3 years. In 2015 and 2016, the mean extent of injury was of moderate impact with values below the 60% at the end of the experiment (day 28; mean ± SE): 29.25% ± 8.19% and 44% ± 8.89% for La Vaca; 28.57% ± 6.55% and 39.64% ± 7.48% for Pota del Llop, and 39.48% ± 7.13% and 46.38% ± 7.22% for Tascons (Figure 2). On the contrary, in 2017, severe impacts with > 60% of average tissue necrosis was observed earlier between days 14 and 16 in all populations. All colonies from La Vaca and Tascons died by day 18 (100% of tissue necrosis). In Pota del Llop, all colonies reached 100% of necrosis by day 24 except for one colony showing > 60% of necrosis (severe impact) at the end of the experiment (Figure 2).

### 3.2 Individual Fitness: Modelling the Response to Thermal Stress

The linear model 2 including the random factor “individual” was retained (lowest statistically significant AIC = 705.21; df = 11 [*χ* ^2^ = 21.6, *p* < 0.001]). The “individual” random factor had a significant effect, suggesting that each individual has a different baseline of resistance to necrosis. Regarding the fixed factors of the model, the deviance test showed that only the “year” factor was significant (Table S2a), while post hoc Tukey test showed that significant differences were due to the year 2017 (Table S2b). The factor “year” was contributing to 89.89% of the variance of the data, followed by the random factor “individual” with a contribution of around 10 times less (9.91%). Finally, “population” and “population‐by‐year” interaction were nonsignificant and showed the lowest contributions: 0.67% and 0.51%, respectively.

### 3.3 Genetic Component: Genetic Structure and Individual Heterosis

**FIGURE 3:**
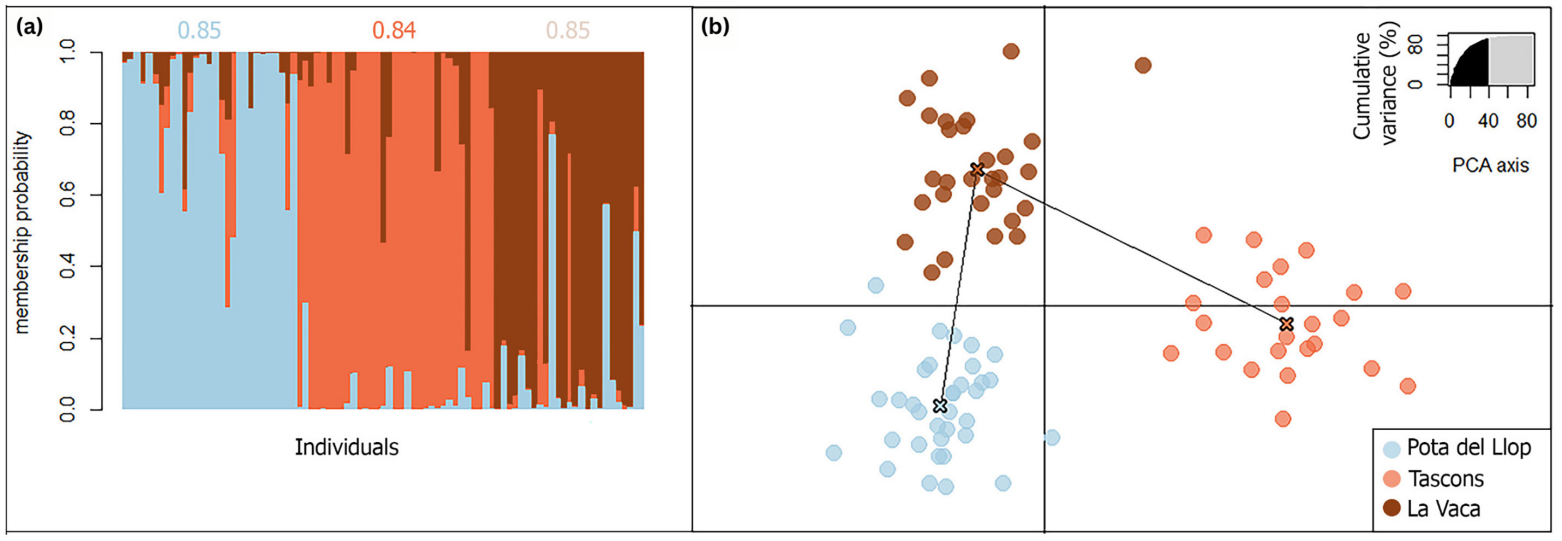
Genetic structure of *Paramuricea clavata* at Medes islands. (a) Individual membership probabilities are represented by a vertical line, where the different color segments indicate the individual proportion to each cluster (*K* = 3) as estimated by the discriminant analysis of principal components (DAPC): Pota del Llop (light blue), Tascons (orange), and La Vaca (brown). Mean membership probabilities are given above each colored cluster. (b) Scatter plot of the DAPC: Each dot corresponds to one individual (*N* = 87) from each of the three populations.

Results on heterozygosity, Hardy–Weinberg equilibrium, allelic richness, and linkage disequilibrium can be found in the Supporting Information (Tables S3 and S4, Figure S3). Three distinct genetic clusters matching the three populations in Medes islands were retrieved from the DAPC analysis with high mean membership probabilities over 84% for each cluster (Figure 3).

Overall, genetic differentiation was low but significant (global *F*_ST_ = 0.015, *p* < 0.001). All pairwise *F*_ST_s were significant: La Vaca versus Pota del Llop (0.013; *p* < 0.001), La Vaca versus Tascons (0.020; *p* < 0.001), Pota del Llop versus Tascons (0.035; *p* < 0.001). The analysis of site‐specific *F*_ST_s suggested that Tascons was the most differentiated population (0.06, 95% HPDI: [0.030; 0.073]), followed by La Vaca (0.04, 95% HPDI: [0.022; 0.059]) and Pota del Llop (0.04, 95% HPDI: [0.021; 0.051]), albeit the differences were not significant (overlapping 95% HPDI; Table S5).

The standard multilocus heterozygosity (sMLH) ranged from 0.49 to 1.48 (Figure S4). The slope of the correlation between sMLH and “nec‐int,” the proxy for individual fitness (random intercepts extracted from linear model 2), was not significant (Monte Carlo permutation test *p* = 0.31, Figures S5, S6 and Table S6).

### 3.4 Environmental Component: Thermal Environments

**FIGURE 4:**
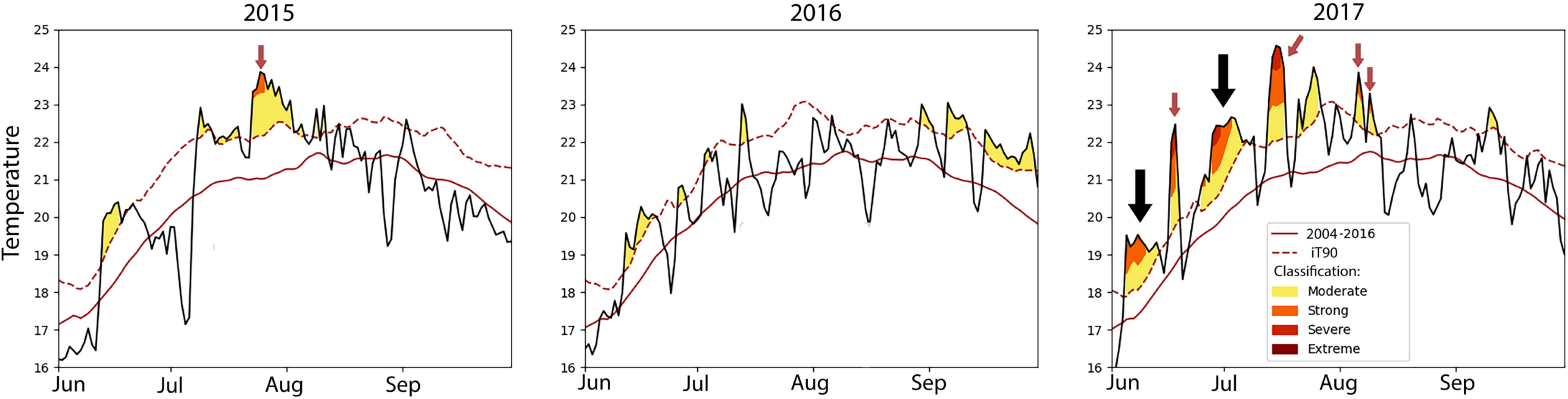
Daily mean temperature values recorded at 15 m from June to September (summer season) at Medes Islands with respect of the interannual climatological mean (red solid line) and 90th percentile (iT90, red dotted line). Days below the red solid line were considered as “cool days,” whereas days above were considered as “warm days.” Following Hobday et al. (2018), marine heatwaves and heat spikes (MHW and MHS) severity classification is as follows: “Moderate” (yellow), “Strong” (orange), “Severe” (red), and “Extreme” (dark red). MHW are highlighted with a black arrow and MHS are highlighted with red arrows. Data taken from the T‐MEDNet initiative (Jou and Ramirez‐Calero 2024).

Recent thermal history patterns, considered as June, July, and August, revealed similar mean ± SD temperatures for the 3 years: 21.3°C ± 1.6°C in 2015, 21.6°C ± 1.0°C for 2016, and 21.8°C ± 1.3°C for 2017 (Table S7). Concomitantly, extreme heat days (T23) were detected in all years during the summer season (Table S7). For 2015 and 2016, at 15 m depth, maximum summer temperatures reached 24.8°C and 23.5°C, respectively, and a low number of total extreme heat days exposure to ≥ 23°C was recorded (*N* = 12 for 2015 and *N* = 2 for 2016; Table S7, Figure S7). The years 2015 and 2016 revealed several periods of anomalous high temperatures during the summer season, but no MHWs (Figure 4, Table S8). Interestingly, the year 2017 reported the highest maximum temperatures of 24.9°C with a total of 19 days of exposure at extreme temperatures (≥ 23°C), surpassing the thermal limit of *P*. *clavata* (Table S7 and Figure S7). In addition, unlike for 2015 and 2016 where no MHW occurred, two MHWs occurred in 2017 with strong, and severe classifications from June to July and several heat spikes (Figure 4). The mean maximum temperature for these MHW was 24.1°C (Table S8).

### 3.5 Plastic Component: “Genotype‐By‐Environment” Interactions and Environmental Sensitivity

**FIGURE 5:**
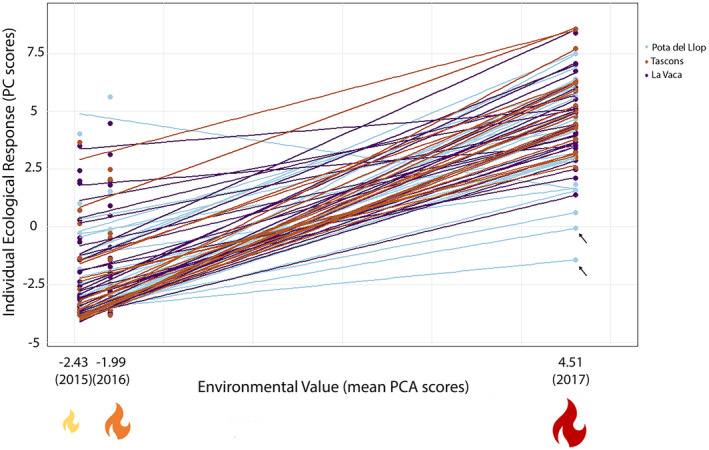
Sensitivity analyses and genotype‐by‐environment interactions of *Paramuricea clavata* during 2015, 2016, and 2017 experiments. Individual ecological response (PC scores) was plotted against environment value for each year/experiment (mean PC score by year). The slope of the regression for each individual is considered as an estimator of the environmental sensitivity of the individual. Populations are displayed in colors: Pota del Llop (light blue), Tascons (brown), and La Vaca (purple).

Regarding the sensitivity analyses, environmental values (yearly mean PCA scores) were −2.43 ± 2.02 for 2015, −1.99 ± 2.22 for 2016, and 4.51 ± 1.96 for 2017. All individuals but one show positive slopes ranging between 0.24 and 1.63. The environmental sensitivity thus varied by a factor of 7 among individuals (Figure 5). Following our classification, all the genotypes were shared among hypersensitive and sensitive categories, including the two genotypes highlighted with arrows (Figure 5). While showing negative PC scores in the three experiments, these two genotypes have a positive regression slope driven by the 2017 experiment. Indeed, they, respectively, reached 100% necrosis by day 24 and severe necrosis impacts (> 60%) at the end of the 2017 experiment. Regarding the significance of the slopes in the null model, a distribution with long tails within a −30 to 30 interval was revealed, which was an undesirable effect of the few data per individual (three data points). Therefore, a statistical test with the standard ∝ level of 0.05 would have been misleading and was thus disregarded. Furthermore, the mean slope of the null distribution was nearly 0 (−0.06), whereas the mean slope of the observed data was 1 with 98.7% of the real data slopes (i.e., per individual) greater than the mean of the null model. As a consequence, there was very limited overlap of the slopes distribution between the null model and the real data (Figure S8). Finally, these results point toward a significantly positive slope for the majority of the individuals.

### 3.6 Field Survey of Necrosis Rates Following 2018 and 2022 MMEs

We encountered 17, 22, and 21 colonies out of the 30 colonies initially marked in Pota del Llop, Tascons, and La Vaca, respectively. Six, three, and five colonies were not impacted (0% of necrosis) in La Vaca, Pota del Llop, and Tascons, respectively (Figure S9a). All the remaining colonies showed recent necrosis (> 70%), albeit with different levels of impact (low, moderate, and severe, Figure S9a). Statistically significant differences were found in average tissue necrosis by population (*F* = 4.295, *p* < 0.018). Pota del Llop displayed the largest percentages of tissue necrosis (30.88% ± 37.22%), followed by Tascons (28.63% ± 32.48%) and La Vaca (7.38% ± 6.25%; Figure S9b). Finally, low impacts (< 30%) were observed in all populations with seven, eight, and fifteen colonies in Pota del Llop, Tascons, and La Vaca (Figure S9a). This latter population did not report additional higher impacts, while severe impacts (> 60% tissue necrosis, including dead colonies) were observed in Pota del Llop and Tascons with four (three dead) and three (two dead) severely impacted colonies, respectively (Figure S9a).

## 4 Discussion

We combined replicated experiments, population genetics, and an in situ field survey to reveal the prominent influence of the environmental component (likely yearly variations in summer thermal regime) on the heterogeneity in ecological responses (i.e., tissue necrosis) to thermal stress in *P*. *clavata* . The low influence of genetic and plastic components combined to the high environmental sensitivity of the tested genotypes point toward a low adaptive potential to recurrent MHWs. This “multiple events” perspective strengthens the recent call to carefully consider predictions on biodiversity evolution based on single‐event experiments.

### 4.1 The Heterogeneity in the Ecological Response to Thermal Stress is Mostly Driven by the Thermal Regime During Summer

The three populations of *Paramuricea clavata* showed high levels of tissue necrosis (moderate to severe mortality) at the end of each experiment confirming the species sensitivity to thermal stress (Crisci et al. 2017; Gómez‐Gras et al. 2022). The ecological responses were similar between populations in 2015 and 2016 (moderate mortality) compared to 2017, in which colonies died before the end of the experiment (severe mortality). Almost 90% (89.89%) of the variance in the ecological response was explained by the factor year (environmental component), while factors individual, population (genetic component), and the population‐by‐year interaction (plastic component) explained only 9.91%, 0.67%, and 0.51%, respectively. Factors individual and year (environmental component) were significant, which suggests different baselines of resistance to thermal stress among individuals and confirms the major environmental effect, mostly driven by the year 2017. This environmental effect was refined by the sensitivity and thermal regime analyses. First, the environmental value (mean phenotype) for 2017 was two to three times higher than the values for 2015 and 2016. Then, we reported positive regression slopes for almost all genotypes supporting an increased negative impact of environmental conditions (environmental sensitivity) from 2015 to 2017. Summer conditions during 2017 showed the largest number of marine heatwaves and heat spikes (MHW and MHSs) compared to 2015 and 2016. Consequently, we posit that colonies of *P*. *clavata* were driven close to their physiological limits by the 2017 extreme summer conditions, which may have hampered any adjustment to thermal stress, whether genetic or plastic, during the experiment.

The relative impact of environmental, genetic, and plastic components in differential responses to ECEs has been screened in different species. For example, ubiquitous population‐by‐environment interactions (plastic component) have been detected in 172 species of plants (Matesanz and Ramírez‐Valiente 2019), but are lacking in others (Shao et al. 2022). Single‐event experiments with tropical corals identified local adaptation (e.g., Thomas et al. 2022) and adaptive plasticity (e.g., Drury et al. 2022) as drivers of differential bleaching responses. Here, we found relatively similar and high levels of necrosis among populations with a prevailing impact of the environmental component. These findings contrast to our previous studies based on “single‐event” experiments at larger spatial scales where differential ecological responses (distinct necrosis levels) were observed among populations (Arizmendi‐Mejía, Linares, et al. 2015; Arizmendi‐Mejía, Ledoux, et al. 2015; Bonacolta et al. 2024; Crisci et al. 2017; Gómez‐Gras et al. 2022). This apparent discrepancy between this and previous studies can be explained considering two nonexclusive hypotheses relying, respectively, (i) on the different spatial scales considered (local scale in the present case vs. regional scale in previous studies) or (ii) on intensification of extreme summer conditions between the first (2009; Crisci et al. 2017) and the last (2017; this study) experimental studies. We discuss these two hypotheses below.

### 4.2 Intraspecific Differences in the Response to Thermal Stress: Does the Geographic Scale Matter or Are Recent Summer Conditions Overwhelming *P*. *clavata* Physiological Capacities?

The experiments conducted to date in *P*. *clavata* have considered a wide range of geographic distances, from local to regional (Arizmendi‐Mejía, Linares, et al. 2015; Crisci et al. 2017) and interregional (Bonacolta et al. 2024; Gómez‐Gras et al. 2022) scales. These experiments demonstrated population heterogeneity in ecological responses to thermal stress triggered by different drivers (e.g., genetic isolation, microorganisms). Considering that the impact of the genetic component on ecological responses can vary over spatial distances (e.g., Galloway and Fenster 2000; Joshi et al. 2001), we hypothesize that the lack of significant population effect observed here can be related to the small spatial scale of the experiment, which flattened the differences between populations. Contrary to previous experiments, we targeted three populations from similar habitats at the same depth range in a close spatial proximity (hundreds of meters). The potential for contrasted genetic make‐up at such small spatial scale is low (but see Ledoux et al. 2015; Richardson et al. 2014) as supported by the comparable levels of genetic isolation of the three populations (overlapping population specific *F*_ST_s). Reanalyzing the different experiments in *P*. *clavata* accounting for ecological or spatial distance between populations should allow to go further in this hypothesis.

Considering a temporal perspective, the discrepancy among experiments in *P*. *clavata* can result from an intensification of ECEs from summer 2009 (Crisci et al. 2017) to summer 2017 (this study), which could have driven the colonies closer to their physiological limits in the later and warmer years. That is, the summer thermal regime previous to the first experiment (summer 2009, Crisci et al. 2017) was less stressful than the 2017 summer thermal regime (this study) allowing some colonies to face the 2009 experimental stress, while colonies were totally swept by the 2017 experimental stress. Three main points support this hypothesis. First, the environmental sensitivity analysis considering 2015, 2016, and 2017 experiments clearly shows an increase of the yearly environmental value between 2015/2016 and 2017. Individuals that showed relatively low necrosis in the first 2 years (negative PC scores) were as strongly impacted in 2017 (positive regression slopes) as individuals showing high necrosis during the first two experiments (regression slopes ~0). Second, necrosis was observed in a vast majority of the marked colonies in the three populations (> 70%) during the field survey following the 2018 and 2022 MHWs. Third, one of the strongest mortality events ever observed was reported in 2022 (Estaque et al. 2023; Rovira et al. b) corroborating the rise in MHWs in the Mediterranean. Strengthening this hypothesis, the intensification of disastrous ECEs in the last decades is not peculiar to the Mediterranean (see Stillman 2019). The frequency of bleaching events in tropical corals increased worldwide since 1980 (Hughes, Anderson, et al. 2018), with detrimental cumulative effects of heatwaves in the last 10 years (Hughes et al. 2021).

### 4.3 What's Next for Shallow Populations of *P*. *clavata* ?

Our results question the persistence of the studied shallow populations of *P*. *clavata* . Both, the experiments and field surveys conducted here supported a limited potential adaptability whether based on genetic or plastic component. Yet, we revealed some variability in the ecological responses at the individual levels as supported by the linear model (significant individual factor explaining 9.89% of the total variance), the sensitivity analyses (environmental sensitivity varies by a factor of 7 among individuals), and the field survey following 2018 and 2022 MMEs. This variability may be seen as a hopeful “raw material” for adaptation to MHWs, particularly when considering that individuals from La Vaca “only” suffered low impacts (10%–30% of tissue necrosis) in the field survey following the two MMEs. Selection acting on the coral holobiont (coral host + microbiome) may promote resistance to thermal stress in these individuals (Bonacolta et al. 2024). However, a complementary field survey in the same region highlighted the global devastating effect of the 2022 MHW on *P*. *clavata* , with almost 70% of surveyed colonies showing tissue necrosis (Rovira et al. 2024). These results point toward a limited adaptive potential of *P*. *clavata* , which is also supported by the contrast existing between the species life history traits (e.g., generation time > 12 years, Coma et al. 1995) and the current temporal dynamics of the MHWs. While dedicated studies to look for potential outlier loci involved in the differential responses to MHWs and to estimate the genomic offset of *P*. *clavata* are needed, any evolutionary responses seem compromised. The potential for adaptation is also questioned in tropical corals in which candidate genetic loci identified to date only show relatively elusive influence on heat stress tolerance (Fuller et al. 2020, but see Matz, Treml, and Haller 2020). In the same collapsing line, the absence of *population‐by‐environment* interactions suggests limited potential for evolutionary changes in adaptive plasticity (Sirovy et al. 2021). Recent studies point toward ecological memory, an increase in stress tolerance following previous exposure, as a key mechanism for coral acclimation to MHWs (Hackerott, Martell, and Eirin‐Lopez 2021; Hughes, Kerry, et al. 2021). Yet, results are contrasting among species with a decrease in bleaching sensitivity following repeated heat stress in some species but not in others (Brown et al. 2023; Hughes et al. 2021). Our study allows first insights into *P*. *clavata* ecological memory. First, colony fragments used in a particular year were submitted to summer thermal conditions of the previous years. Yet, the worst ecological responses to thermal stress were observed during the last experiment in 2017 with high environmental sensitivity (positive slopes and lack of resistant genotypes in the sensitivity analyses). Then, most marked colonies showed necrosis during field surveys following 2018 and 2022 MHWs events. These results question any increase in thermotolerance as expected with the ecological memory hypotheses and strengthen the limited potential for adaptation in *P*. *clavata* .

## 5 Conclusion

As temperature and frequency of ECEs continue to rise (Hughes et al. 2021; Garrabou et al. 2022), the reduction of biodiversity is more than ever a central concern for society. Adopting a “multiple events” perspective that combined replicated common‐garden experiments in aquaria and mortality surveys in the field performed on the same colonies, our study points toward a potential collapse of many of the shallow populations of *P*. *clavata* . This collapse would emerge from a low to nonexistent adaptive response, whether driven by genetic adaptation or plasticity, combined to a high environmental sensitivity and a potential intensification of MHWs. Considering the small spatial scale of our study, projection at larger scale should be made cautiously. Yet, population collapses of *P*. *clavata* linked to recurrent MHWs have been observed in other Mediterranean regions (Cupido et al. 2009; Garrabou et al. 2021; Gómez‐Gras, Linares, López‐Sanz, et al. 2021; Ruffaldi Santori et al. 2021). Moreover, field surveys following the 2022 MHW event in this and in other regions reported terrific mortality rates (Rovira et al. 2024). Hundreds of km apart, populations above 20 m depth displayed on average > 80% of affected colonies and an increase by 142% of the degree of impact following the 2022 MHW compared to the previous 2003 MHW (Estaque et al. 2023). Worrying, this trend in *P*. *clavata* could likely be transposable to many of the Mediterranean habitat‐forming and sessile species impacted by MHWs (Garrabou et al. 2022; Gómez‐Gras, Linares, Dornelas, et al. 2021). We thus predict a shift in these species' upper distribution limits, which will lead to a simplification of associated benthic communities hampering potentially related ecosystem functions and services (Gómez‐Gras, Linares, Dornelas, et al. 2021).

This study echoes two recent calls regarding the future of marine diversity in the context of ECEs. First, the impacts of ECEs on biodiversity should be studied from a temporal perspective, which accounts for their recurrence (Hughes et al. 2021). In this line, current models of biodiversity evolution based on “single‐event” approaches will benefit from studies using a “multiple events” perspective. Then, while conservation and restoration actions should be able to slow locally the collapsing trend of marine habitat‐forming species (Zentner et al. 2023) by managing local stressors (e.g., pollution), immediate action on greenhouse gas emissions remains the only way to protect these species globally.

## Author Contributions

**Sandra Ramirez‐Calero:** conceptualization, data curation, data analysis, writing original draft. **Daniel Gómez‐Gras:** experimental setup, data collection, write – review and editing. **Aldo Barreiro:** data analysis and validation, write – review and editing. **Nathaniel Bensoussan:** data collection and validation, review and editing. **Laura Figuerola‐Ferrando:** data collection, experimental setup, review and editing. **Marc Jou:** data visualization, review and editing. **Àngel López‐Sanz:** data collection, experimental setup, review and editing. **Paula López‐Sendino:** data collection, experimental setup, review and editing. **Alba Medrano:** data collection, experimental setup, review and editing. **Ignasi Montero‐Serra:** data collection, experimental setup, data analysis, review and editing. **Marta Pagès‐Escolà:** data collection, experimental setup, review and editing. **Cristina Linares:** data collection, review and editing, funding acquisition. **Jean‐Baptiste Ledoux:** conceptualization, data collection, experimental setup, data analysis, write – review and editing, funding acquisition. **Joaquim Garrabou:** conceptualization, data collection, experimental setup, data analysis, write – review and editing, funding acquisition.

## Conflicts of Interest

The authors declare no conflicts of interest.

## Supporting information


Data S1.

## Data Availability

The data and code that support the findings of this study are openly available from Zenodo at https://doi.org/10.5281/zenodo.13959703 and GitHub at https://github.com/sandrarcr/Pclavata_rec_MHW.git (in‐situ temperature data, ecological data, linear models and genetic structure analysis) and in Zenodo at https://doi.org/10.5281/zenodo.14007188 and GitHub at https://github.com/Damyck/tMednet.git (detection and analysis of MHWs in the SST data). Microsatellite loci information is available from the Molecular Ecology Resources Database under accession numbers GU386255–GU386265 and PQ513430–PQ513439 and GeneBank at https://10.1111/j.1755‐0998.2010.02871.x.
